# Cognitive impairment and vulnerability of cholinergic brain network in the Alzheimer’s continuum: free-water imaging based on diffusion tensor imaging

**DOI:** 10.3389/fnins.2025.1587702

**Published:** 2025-04-30

**Authors:** Simin Zhao, Yuting Nie, Lulu Wen, Xinzuo Qin, Liyuan Huang, Changbiao Chu, Miao Qu

**Affiliations:** ^1^Third Clinical Medical College of Beijing University of Chinese Medicine, Beijing, China; ^2^The Department of Neurology, Gansu Provincial Hospital, Lanzhou, China; ^3^Department of Neurology, Xuanwu Hospital of Capital Medical University, Beijing, China; ^4^Department of Chinese Medicine, Xuanwu Hospital of Capital Medical University, Beijing, China

**Keywords:** diffusion tensor imaging, free water imaging, cognitive impairment, cholinergic brain network, Alzheimer’s disease

## Abstract

**Background:**

Increased extracellular free water (FW) is considered to provide better pathophysiological information than conventional diffusion tensor imaging (DTI) metrics. The cholinergic brain network is a key hub for cognitive function, and microstructural changes detected by free water imaging in this system may be associated with cognitive impairment in Alzheimer’s disease (AD). However, the specific impact of FW changes in the cholinergic brain network on cognitive domains across the AD continuum and their diagnostic value remain unclear.

**Methods:**

Here, we investigated the basal forebrain cholinergic free water alterations based on free water-corrected diffusion tensor imaging in healthy controls (*n* = 36), amnestic mild cognitive impairment (aMCI; *n* = 31), the AD group (*n* = 33). The cholinergic basal forebrain subregions were divided into the Broca diagonal band (Ch1-3) and the Meynert basal nucleus (Ch4). The cognitive domains performance was measured using the Montreal Cognitive Assessment (MoCA). Additionally, we evaluated the diagnostic value of free water fraction (FWf) within the cholinergic system.

**Results:**

FWf in the bilateral Ch1-3 and Ch4 regions increased with age, and was significantly higher in aMCI and AD (*p* < 0.001). In AD, the FWf within Ch4 was correlated with total MoCA score (*R* = −0.42, *p* = 0.015), especially with visual spatial/executive (*R* = −0.47, *p* = 0.006) and orientation deficits (*R* = −0.38, *p* = 0.029). No significant correlations were found in the aMCI group. ROC curve analysis showed that FWf within the cholinergic brain network had high diagnostic efficacy for AD versus HC (AUC = 0.958, 95% CI = 0.909–1.00), and moderate diagnostic efficacy for aMCI versus HC (AUC = 0.795, 95% CI = 0.685–0.905) and aMCI versus AD (AUC = 0.719, 95% CI = 0.589–0.850).

**Conclusion:**

FW imaging captures microstructural damage in the cholinergic brain network across the entire AD continuum. These changes occur early in aMCI but selectively affect domain-specific cognition in the later stages of AD, possibly through cholinergic network dysfunction. Our results highlight the potential of free water imaging as a biomarker for cognitive decline.

## Introduction

Alzheimer’s disease (AD) is the leading cause of dementia, primarily affecting memory, while other cognitive domains such as orientation, executive function, and language abilities may also be impaired. It is projected that by 2050, the global burden of AD will be substantial, with an estimated 139 million individuals affected worldwide (Jia et al., 2020; Alzheimer's Association, 2020). Amnestic mild cognitive impairment (aMCI) represents an early and critical precursor to AD (Du et al., 2021; Petersen, 2004). Unlike other subtypes of MCI, which may be associated with various forms of dementia, aMCI is distinguished by its high sensitivity and specificity in predicting the progression to AD (Mitchell and Shiri-Feshki, 2009). Therefore, the pathological pathways underlying cognitive changes in AD may already be observable in aMCI, providing a unique opportunity to investigate the mechanisms of cognitive decline across the AD continuum. A recent study has demonstrated that cholinergic pathways exhibit alterations in the early stages of the AD continuum and are associated with cognitive performance (Nemy et al., 2023).

AD has been established as a neurodegenerative disorder characterized by dysfunction of the cholinergic system (Bohnen et al., 2018). The cholinergic basal forebrain (cBF) network is a subcortical network located in the frontal lobes subcortex, mainly composed of cholinergic neurons, including several key components: the medial septal nucleus (Ch1), the vertical and horizontal limbs of the diagonal band of Broca’s nucleus (Ch2 and Ch3), and the extensive cellular complex (Ch4; nucleus basalis of Meynert) (Mesulam et al., 1983), playing a key role in regulating cortical acetylcholine levels and cognitive function. Impairment of the cBF system is closely associated with cognitive decline, which has been extensively studied in AD (Grothe et al., 2014; Schumacher et al., 2023). Previous research has demonstrated that severe neuronal damage in the cBF of AD patients leads to both cognitive and behavioral deficits, accompanied by a 90–95% reduction in cortical acetylcholine activity (Ballinger et al., 2016; Geula et al., 2021). Notably, AD is a form of dementia characterized by multi-domain cognitive impairment, with memory being the most prominently affected domain. The neurons within hippocampus and cBF are selectively vulnerable to AD pathology (Fu et al., 2018). While hippocampal involvement is linked to memory decline, the relationship between the cBF and its subregions and specific cognitive domains remains unclear. Recent studies demonstrate that cBF subregions (e.g., Ch1/2 and Ch4) exhibit distinct cognitive associations: Ch1/2 atrophy correlates with episodic memory deficits in preclinical AD (Scheef et al., 2019), while Ch4 degeneration predicts attention and visuospatial decline in MCI and synucleinopathies (Brueggen et al., 2015; Rémillard-Pelchat et al., 2022), highlighting its domain-specific role beyond hippocampal memory circuits.

Currently, the free water (FW) imaging technique derived from diffusion-weighted imaging (DWI) can be used to assess neurodegenerative changes or neuroinflammatory response in microstructural regions such as the cBF, and it is emerging as an important tool for the development of biomarkers of neurodegenerative diseases (Schumacher et al., 2023; Wu et al., 2024). FW imaging estimates extracellular water content and has emerged as a novel tool for identifying disease-related changes in the brains of patients with neurodegenerative disorders, including Parkinson’s disease (PD) and AD (Schumacher et al., 2023; Ray et al., 2023). It has also been proposed as a potential biomarker for neurodegenerative changes. Previous studies have demonstrated that FW in the cBF can help identify PD patients with cognitive impairment (Ray et al., 2023) and is associated with attention/working memory and executive function (Crowley et al., 2024). Another study revealed that FW in the cortical pathways of the cBF is abnormally increased in both dementia and MCI patients and correlates with cognition (Nemy et al., 2023). However, it remains unclear whether the impact of FW changes in different subregions of the cBF on specific cognitive domains varies across the AD continuum.

In this study, we aim to investigate FW changes in subregions of the cBF using DWI imaging. By including individuals with aMCI and AD, we seek to examine changes in FW across the AD continuum and explore how these microstructural alterations are linked to different cognitive domains at various disease stages.

## Methods

### Study participants

Participants for this research were enrolled from Xuanwu Hospital, which is affiliated with Capital Medical University, and the Guang’anmennei Community Health Service Center. The study protocol was approved by the Ethics Committee of Xuanwu Hospital of Capital Medical University [Approval No. (2020) 097] and adhered strictly to the ethical principles of the Declaration of Helsinki. The study has been registered on the Chinese Clinical Trial Registry (ChiCTR2100041801). After being thoroughly informed about the study’s details and potential consequences, participants and their legal representatives willingly provided their signatures on consent forms.

A total of 100 subjects participated in the study, comprising 33 patients with AD, 31 patients with aMCI and 36 HC. Each participant was right-handed and had a head MRI as well as neuropsychological assessments. Patients with AD were selected in accordance with the 2011 diagnostic criteria established by the National Institute of Aging-Alzheimer’s Association (NIA-AA) for clinical identification of the condition (McKhann et al., 2011). The aMCI was also diagnosed based on the NIA/AAMCI core criteria, by two experienced neurologists, all patients with aMCI had Montreal Cognitive Assessment (MoCA) score below the lower limit of normal and clinical dementia rating scale (CDR) score of 0.5 (including a mandatory memory score of 0.5) (Albert et al., 2011). Healthy controls matched for the age and sex distribution of aMCI patients were selected, and scores in MoCA and CDR assessments should be within normal limits and no complaints of memory loss were reported. The exclusion criteria were as below: (1) with a history of significant depression or other mental illnesses (such as schizophrenia, bipolar disorder, or mania); (2) with drugs or alcohol abuse during the previous two years; (3) with hypothyroid, severe infection, deficit of vitamin B12 and folic acid, or inflammatory encephalopathy, (4) with malignant tumors within three years; (5) with history of intracranial organic lesions, infection, cerebral trauma, or surgery; and (6) with long-term steroid hormone administration.

### Demographic and cognitive assessment

The present investigation applied a standardized methodology to ensure the homogeneity and comparability of the data. The data on gender, age, and educational background were recorded, as these factors have been widely acknowledged as significant confounders affecting cognitive function. A self-administered questionnaire was used to investigate the participants’ daily habits, including their alcohol consumption, smoking and tea consumption. A thorough evaluation of the participants’ health status was carried out, gathering comprehensive details on the underlying disease.

The implementation of the cognitive assessment was performed by professionally trained and certified assessors to ensure the accuracy and reliability of the assessment. In this study, we used MoCA to assess participants’ cognitive function. The Beijing version of MoCA was selected, which is widely used in mainland China and generally showed good internal consistency and corresponding standard validity (Yu et al., 2012). It consists of seven cognitive domains, namely visuospatial/executive function, naming, attention, abstraction, language, delayed memory, and orientation.

### MRI data acquisition

MRI data were acquired using a 3.0 T Siemens scanner equipped with a 16-channel phased-array head–neck coil. Noise was minimized using earplugs and headphones, and foam pads were placed on both sides of the head to reduce head movement. The parameters of the diffusion tensor imaging (DTI) acquisition were as follows: FOV: 224 mm, matrix size: 128 × 128, slice thickness: 2 mm, number of slices: 60, repetition time: 8600 ms, echo time: 99 ms, number of diffusion direction: 64, b value: 0 and 1000 s/mm^2^. In the MRI data, participants with excessive head movement or metal artifacts were excluded. In addition, routine MRI scans, including 3D T1 and T2 scans, were performed to screen for organic brain abnormalities.

### Preprocessing of the DTI data

Initially, the conversion of raw DTI data into the NIFTI format was accomplished using the MRIcroN tool. Concurrently with this conversion, the necessary b vector and b value files were produced for the subsequent processing of DTI data. Subsequently, the pre-processing of these DTI images was carried out utilizing the FMRIB Software Library version 6.0.^1^ Data preprocessing encompassed a series of systematic steps: First, we corrected DTI data for each subject using FSL’s “eddy_correct” tool to mitigate distortions attributable to eddy currents and motion. Subsequently, the original b-vectors were rotated to align with the post-correction data using the “fdt_rotate_bvecs” tool. Extraction of b0 images was then conducted. Thereafter, brain extraction was performed on the b0 images utilizing the “bet2” tool. Finally, diffusion tensor fitting was executed with the “dtifit” tool.

### Free water analysis and regions of interest selection

**Figure 1:**
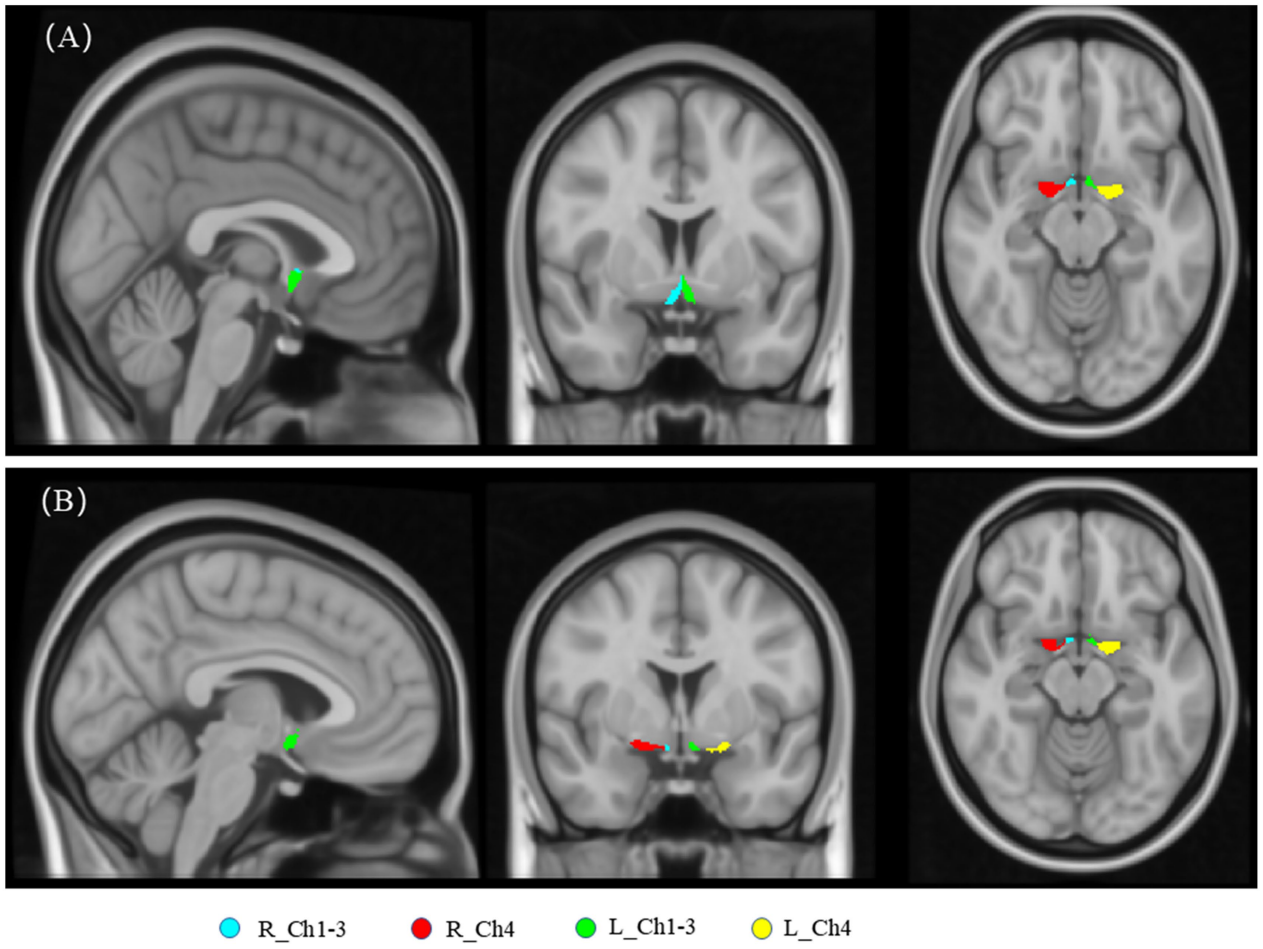
The basal forebrain cholinergic regions at distinct anatomical levels are illustrated in **(A)** and **(B)**. R_Ch1-3: Right Vertical and horizontal limbs of the diagonal band of the medial septal nucleus, Broca’s nucleus (Ch1, Ch2, Ch3). R_Ch4: Right Meynert basal nucleus (Ch4). L_Ch1-3: Left Vertical and horizontal limbs of the diagonal band of the medial septal nucleus, Broca’s nucleus (Ch1, Ch2, Ch3). L_Ch4: Left Meynert basal nucleus (Ch4).

Utilizing the regularized two-tensor model and the Diffusion Imaging in Python (Dipy) software package, fiber water (FW) images were derived from the preprocessed diffusion-weighted imaging data^2^ (Garyfallidis et al., 2014). The selection of region of interest (ROI) was based on a probabilistic map developed from deceased individuals (Zaborszky et al., 2008). This map was implemented in the JuBrain Anatomy Toolbox version 2.2 (Eickhoff et al., 2005). According to the Mesulam naming system (Mesulam et al., 1983), specific areas of cholinergic base forebrain were selected as masks including: the bilateral Ch4 ROI, which corresponds to the Meynert basal nucleus, and bilateral Ch1-3 ROI, which involve the medial, vertical and horizontal branches of the Broca diagonal zone (as shown in Figure 1).

The tract-based spatial statistics (TBSS) skeleton projection procedure was employed to analyze the FW imaging (Andica et al., 2019). The steps were as follows: Firstly, the fractional anisotropy (FA) maps of all subjects were aligned to the standard Montreal Neurological Institute (MNI) space (MNI152) using the FMRIB’s Nonlinear Image Registration Tool. Based on this normalized data, the FA threshold was set at 0.2. Subsequently, a group-averaged FA map was further generated and thresholded to construct an average FA skeleton that mapped the centers of the common fiber tracts of the group. Finally, the FW maps of individual participants were projected onto the average FA skeleton to obtain the skeletonized FW maps for each participant. The fslmeants script was used to extract the FW values of each participant along the skeleton template, generating a numerical matrix.

### Statistical analyses

Continuous variables that follow a normal distribution are reported as the mean along with their standard deviations, while categorical variables are expressed in terms of frequency distributions. For continuous variables exhibiting non-normal distributions, the median along with the 25th and 75th percentiles are reported. To compare baseline characteristics across various participant groups for continuous variables that are normally distributed, an initial Analysis of Variance (ANOVA) is conducted. When dealing with categorical variables, the Chi-square test is the standard approach; however, if the expected frequency count is less than five, Fisher’s exact test is preferred. For the assessment of non-normally distributed data across three groups, the Kruskal-Wallis test is implemented.

Spearman’s correlation was used to quantify the associations between the FW value and age. The 2-sample *t*-test was used to compare the gender differences in the FW value. The differences in the FW value of the bilateral Ch1-3 and Ch4 regions between two groups were evaluated using a general linear model univariate while controlling for age, gender, and years of education. A two-tailed significance level of 0.05 was set to define statistical significance. Additionally, the correlations between the FW value within the cBF and total MoCA scores and MoCA domain scores (7 items) were examined through Spearman’s correlation analysis, with adjustments made for education, age, and gender. Since each region is correlated with multiple scores (*n* = 8), multiple-test correction is required. We used the Bonferroni method for multiple-test correction and set the threshold of *p* < 0.05/8 as the threshold for significant statistical significance, while 0.05/8 < *p* < 0.05 was defined as suggestive statistical significance. Furthermore, the determination of potential biomarkers is facilitated by the application of Receiver Operating Characteristic (ROC) curve analysis. All statistical analyses were conducted using IBM SPSS software (version 25.0; https://www.ibm.com/spss) and R (version 4.1.2; https://www.r-project.org).

## Results

### Demographic and clinical characteristics

As shown in Table 1, we matched for age, sex, and educational level to ensure that the three groups were comparable on these demographic variables (*p* > 0.05). Furthermore, lifestyle factors, including smoking and drinking, as well as chronic disease status, such as hypertension, diabetes mellitus, hyperlipidemia (HDM), cerebral infarction, coronary heart disease, and thyroid disease, showed no significant differences among the three groups (all *p* ≥ 0.05). The total MoCA score and MoCA domain scores exhibited significant differences among the three groups (all *p* < 0.001).

**Table 1:** Demographics analysis and cognitive performance.

Characteristic	HC (*n* = 36)	aMCI (*n* = 31)	AD (*n* = 33)	*p*-values
Age (year)	65.25 ± 6.23	68.71 ± 8.19	68.485 ± 6.53	0.076
Male, *n* (%)	8 (22.2%)	8 (25.8%)	14 (42.4%)	0.155
Education (year)	10.6 (9, 12)	12 (9, 12)	9 (5.5, 11.5)	0.050
Current smoking, *n* (%)	2 (5.6%)	4 (12.9%)	8 (24.2%)	0.095
Current drinking, *n* (%)	4 (11.4%)	4 (12.9%)	6 (18.2%)	0.719
Current Tea Drinking, *n* (%)	21 (57.1%)	13 (41.9%)	12 (36.4%)	0.162
Hypertension, *n* (%)	10 (27.8%)	10 (32.3%)	8 (24.2%)	0.775
Diabetes mellitus, *n* (%)	9 (22.9%)	3 (9.7%)	6 (18.2%)	0.266
Hypercholesterolaemia, *n* (%)	7 (19.4%)	6 (19.4%)	2 (6.1%)	0.201
Previous cerebral infarction, *n* (%)	3 (8.3%)	3 (9.7%)	3 (9.1%)	1.000
Coronary artery disease, *n* (%)	3 (8.3%)	3 (9.7%)	2 (6.1%)	0.903
Thyroid Diseases, *n* (%)	1 (2.9%)	1 (3.2%)	2 (6.1%)	0.836
MoCA score	26 (25, 27)	23 (21, 25)	12 (10, 18)	<0.001
Visuospatial/executive	4 (4, 5)	4 (3, 5)	2 (1, 4)	<0.001
Naming	3 (3, 3)	3 (3, 3)	3 (2, 3)	<0.001
Attention	6 (5, 6)	5 (4, 6)	4 (2, 4.5)	<0.001
Language	2 (2, 3)	2 (1, 2)	1 (0, 1.5)	<0.001
Abstraction	2 (1, 2)	1 (1, 2)	0 (0, 1)	<0.001
Delayed recall	3 (2, 4)	1 (0, 3)	0 (0, 0)	<0.001
Orientation	6 (6, 6)	6 (5, 6)	3 (1, 4)	<0.001

In the present study, comparisons of continuous variables among the three groups were performed using one-way analysis of variance (ANOVA), followed by Bonferroni’s post hoc multiple comparison test. For categorical variables, chi-square tests were employed, and when the expected count was less than 5, Fisher’s exact test was utilized. Non-normally distributed data across the three groups were compared using the Kruskal-Wallis test, with post hoc analysis conducted using the Steel-Dwass test for pairwise comparisons.

### The free-water value feature and between-group differences

**Figure 2:**
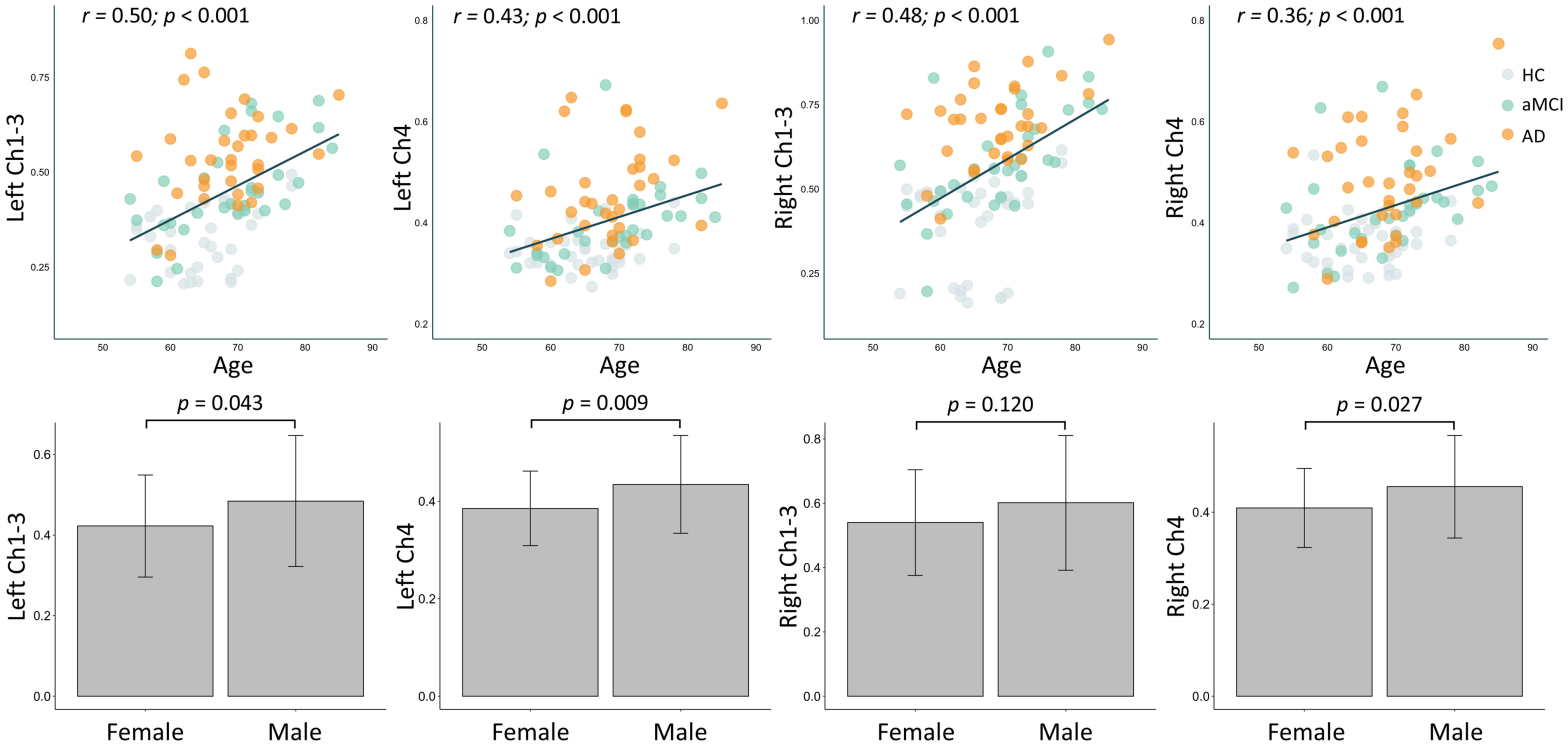
Effects of age and gender on the free water within the cholinergic system of the basal forebrain in all participant.

**Figure 3:**
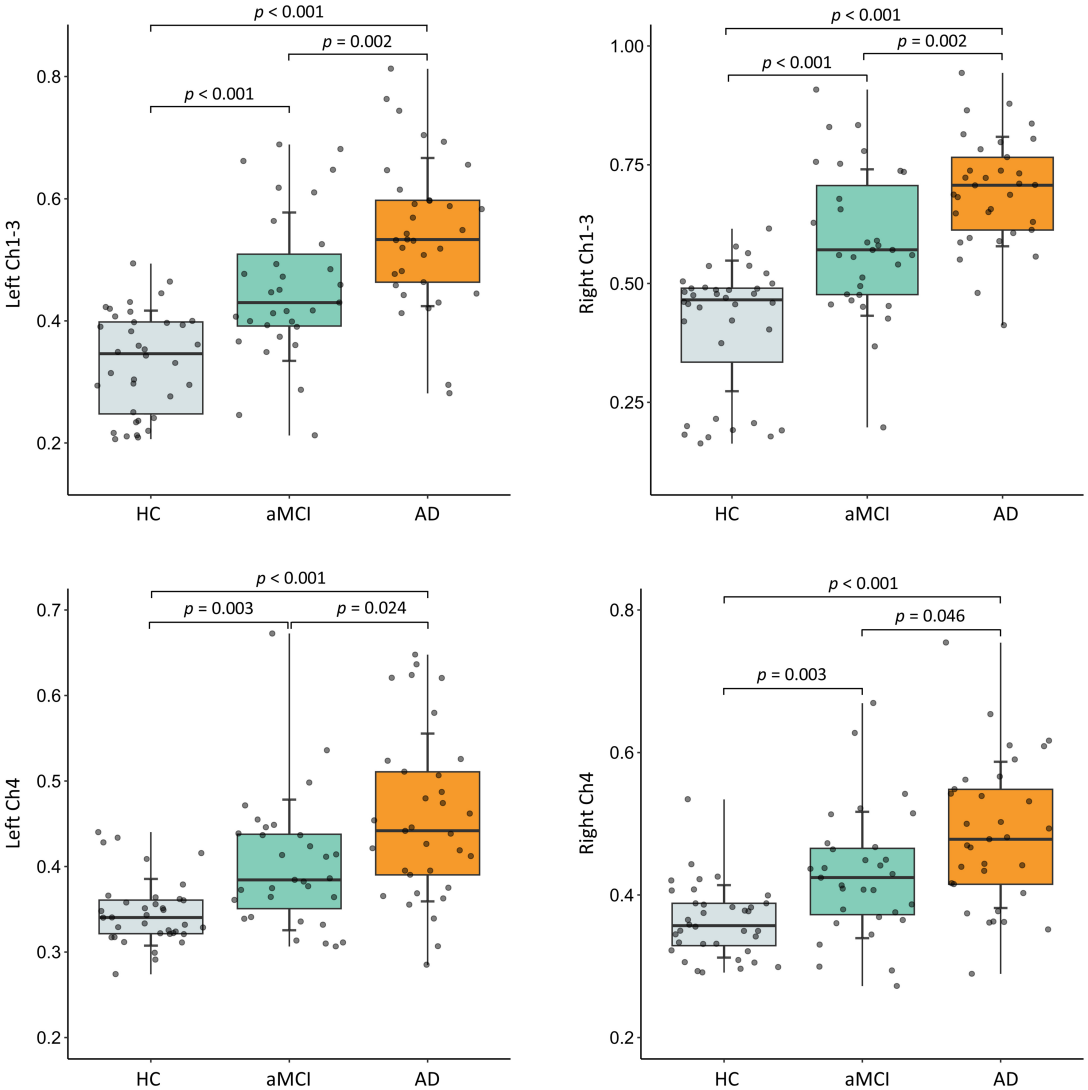
Between-group differences in free water in bilateral Ch1-3 and Ch4 regions of the basal forebrain cholinergic system. HC, healthy controls; aMCI, amnestic mild cognitive impairment; AD, Alzheimer’s disease. The *p*-value has been adjusted for age, gender, and years of education.

As shown in Figure 2, the FW values in the bilateral Ch1-3 and Ch4 regions all increased with age (all *R* > 0.3, *p* < 0.001). Additionally, the FW values in the left Ch1-3, left Ch4, and right Ch4 regions were significantly higher in males than in females (all *p* < 0.05), while there was no difference in the right Ch4 region (*p* = 0.120). As shown in Figure 3, after adjusting for age, sex, years of education, the FW values in the bilateral Ch1-3 and Ch4 regions of patients with aMCI and AD were higher than those of healthy controls (all *p* < 0.05). Compared with aMCI patients, the FW values in these regions of AD patients were even higher (all *p* < 0.05).

### Correlation between cognitive performance and free-water value

**Figure 4:**
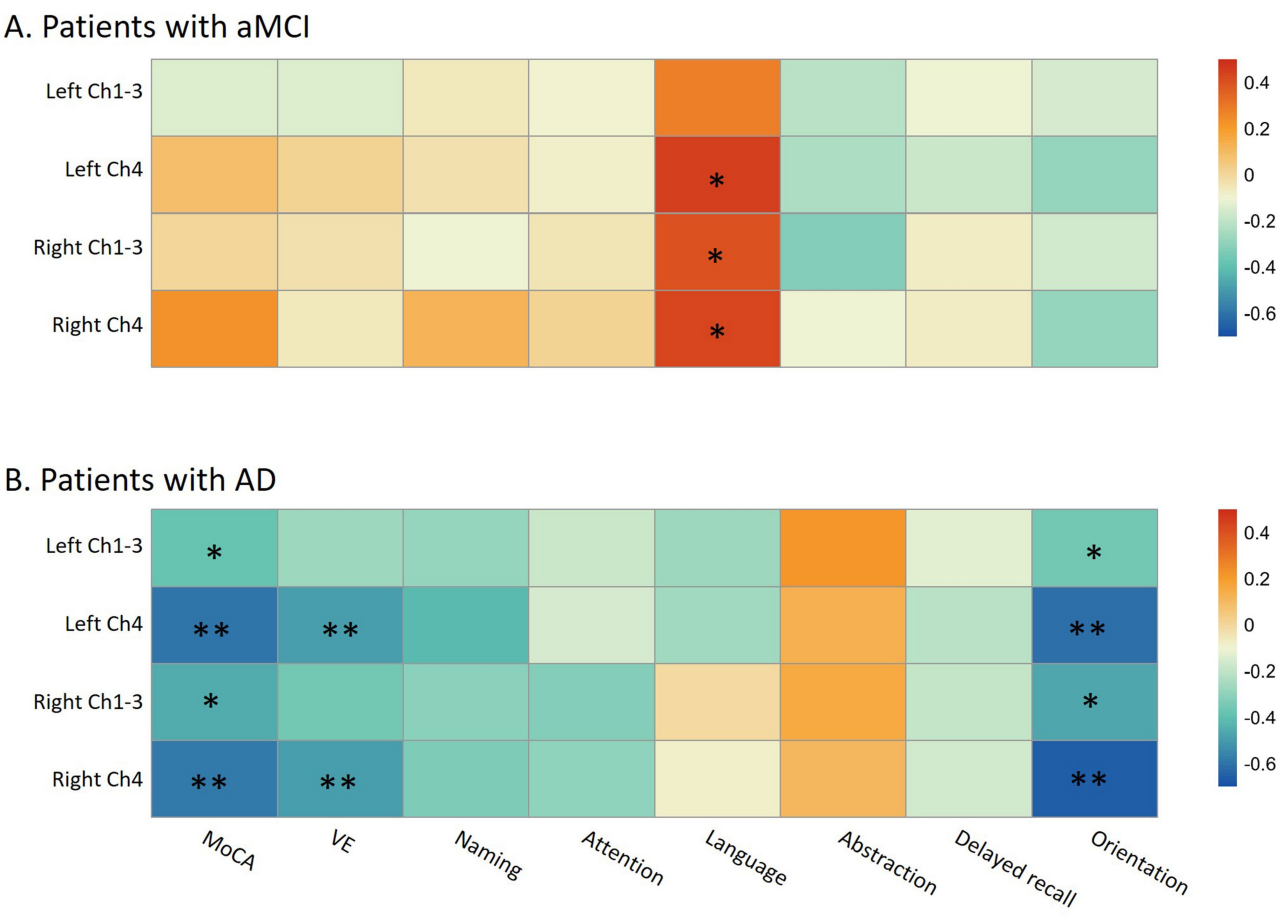
The relationship between cognitive performance and free-water in bilateral Ch1-3 and Ch4 regions of the basal forebrain cholinergic system in aMCI patients **(A)** and AD patients **(B)**. aMCI, amnestic mild cognitive impairment; AD, Alzheimer’s disease; MoCA, Montreal Cognitive Assessment; VE, visuospatial/executive. In the heatmap, the color is related to the magnitude of the Spearman’s correlation coefficient, which has been adjusted for age, gender, and years of education. MoCA consists of seven cognitive domains, namely visuospatial/executive function, naming, attention, abstraction, language, delayed memory, and orientation. * *p* < 0.05 (suggestive evidence); ** Indicates that the *p*-value has passed the Bonferroni multiple-test correction (*p* < 0.05/8; significant evidence).

In patients with aMCI, no correlation was observed between the FW values in the bilateral Ch1-3 and Ch4 regions and the total MoCA score (all *p* > 0.20). Suggestive evidence indicated that the FW values in the left Ch4 (*R* = 0.45, *p* = 0.017), right Ch4 (*R* = 0.43, *p* = 0.022), and right Ch1-3 regions (*R* = 0.40, *p* = 0.036) were positively correlated with the language domain score of MoCA. However, this correlation did not withstand multiple-test correction (Figure 4A). In patients with AD, suggestive evidence suggested a negative correlation between the FW values in the bilateral Ch1-3 regions and both the total MoCA score (*R* = −0.38 ~ −0.45, *p* = 0.036~0.012) and the orientation domain score of MoCA (*R* = −0.36 ~ −0.47, *p* = 0.048~0.009). Nevertheless, this correlation did not remain significant after multiple-test correction (0.0063 < *p* < 0.05). Additionally, significant evidence demonstrated that the FW values in the bilateral Ch4 regions were negatively correlated with the total MoCA score (*R* = −0.60 ~ −0.59, *p* = 0.0004~0.0006), as well as the visuospatial/executive (*R* = −0.50 ~ −0.49, *p* = 0.005~0.006) and orientation (*R* = −0.65 ~ −0.61, *p* = 0.0003~0.0001) domain scores of MoCA (all *p* < 0.0063; Figure 4B).

### Diagnostic efficacy of basal forebrain cholinergic system FWf within predicting aMCI

**Figure 5:**
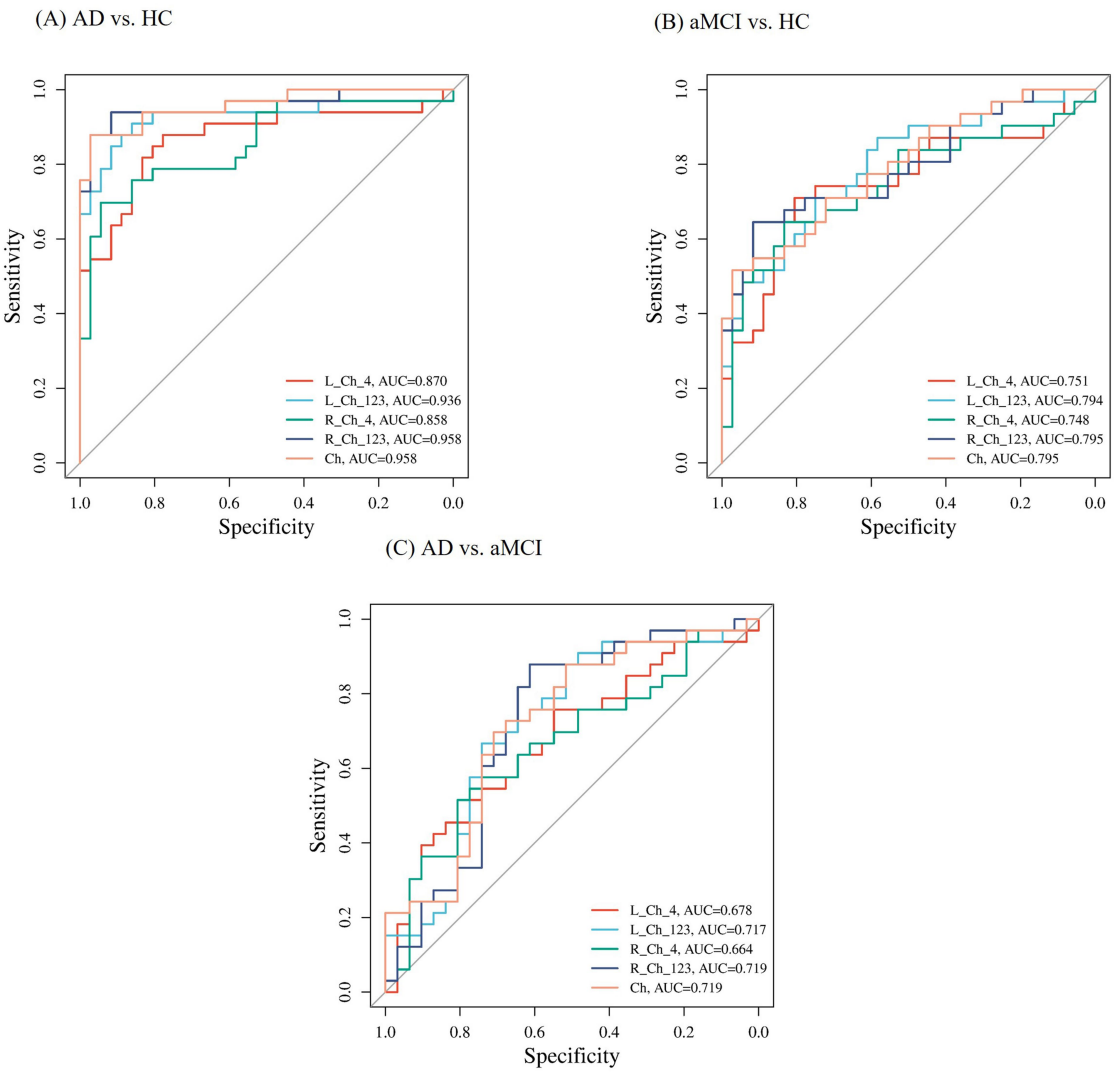
The subject operating characteristic curve (ROC) used to distinguish between AD, aMCI and HC subjects. **(A)** Represents the ROC curve comparing AD patients with HC. **(B)** Depicts the ROC curve for aMCI individuals in contrast with healthy controls HC. **(C)** Illustrates the ROC curve for AD patients in comparison with aMCI patients.

As shown in Figure 5, based on the ROC curve, we evaluated the FWf within the cBF for predicting the patients with aMCI and AD. In distinguishing the FWf within the cBF between the NC group and the patients with AD, the AUC areas for FWf in the Left_Ch1-3, Left_Ch4, Right_Ch1-3, and Right_Ch4 region were 0.936 (0.875, 0.997), 0.87 (0.778, 0.961), 0.958 (0.909, 1), and 0.858 (0.766, 0.949), respectively. The combined diagnostic AUC area for FWf across the four Ch regions was 0.958 (0.909, 1).

In distinguishing the FWf within the cBF between the NC group and the patients with aMCI, the AUC areas for FWf in the Left_Ch1-3, Left_Ch4, Right_Ch1-3, and Right_Ch4 regions were 0.794 (0.686, 0.902), 0.751 (0.628, 0.874), 0.795 (0.685, 0.905), and 0.748 (0.625, 0.872), respectively. The combined diagnostic AUC area for FWf across the four Ch regions was 0.795 (0.685, 0.905).

Subsequently, we assessed the diagnostic efficacy of FWf within the basal forebrain cholinergic system for predicting the patients with aMCI and AD, and found that the AUC areas for FWf in the Left_Ch1-3, Left_Ch4, Right_Ch1-3, and Right_Ch4 regions were 0.717 (0.588, 0.847), 0.678 (0.546, 0.810), 0.719 (0.589, 0.850), and 0.664 (0.529, 0.798), respectively. The combined diagnostic AUC area for FWf across the four Ch regions was 0.719 (0.589, 0.850).

## Discussion

In this research, we explored the alterations of basal forebrain cholinergic free water (FW) based on free water-corrected diffusion tensor imaging in the AD continuum, and further evaluated the associations with the performance of cognitive domains. Our study found that an increase in FW in sub-regions of the cBF occurred in both AD and aMCI, with higher levels in AD than in aMCI, suggesting that FW changes in cBF may occur in the early clinical stage of AD. Although the increase in cBF was present in the aMCI population, it was not associated with the performance of cognitive domains. In contrast, in the AD population, the FW alterations in the Ch4 sub-region of cBF were related to overall cognition, as well as visuospatial/executive and orientation functions. Additionally, we also found that the FW of cBF increased with age, and males seemed to have higher FW levels. These results indicate that the FW changes in cBF emerges in the early stage of AD, but mainly affects specific cognitive functions in the late stage of the disease.

Our study revealed that, in comparison to healthy controls, the AD continuum had significantly increased FW within the bilateral basal forebrain cholinergic regions (bilateral Ch1-3 and Ch4), and this increase occurred at an early stage and gradually rose with disease progression. Our study suggested that the FW in the cBF region could serve as an important early biomarker for AD and be used to monitor disease progression. Our study was supported by previous research. Compared to the control group, the dementia and MCI groups both exhibited an increase in FW within the Ch4-cortical cholinergic pathway (Schumacher et al., 2023). Conventional volumetric measures like hippocampal volume were less effective than the integrity of the cholinergic pathways in identifying the early stages of AD (Nemy et al., 2023). Furthermore, the results of our study were in line with previous cross-sectional studies using various techniques, such as basal forebrain volume measurement (Muth et al., 2010), structural covariance network (Fu et al., 2021), and Aβ load (Kerbler et al., 2015), which all highlighted the degradation of the cholinergic system in people with AD. Early monitoring of FW in the cBF region may contribute to the identification of MCI patients who convert to AD. However, follow-up studies are still needed to further clarify the relationship between the alterations of FW in cBF and AD-specific biomarkers and pathology.

The FW imaging had shown significant potential in identifying brain microstructural alterations, which was often regarded as a hallmark feature of neurodegenerative changes. The mechanisms by which it is involved in the pathogenesis of AD may be related to multiple mechanisms. Some studies have suggested that FW can be used to monitor the early changes in the pathophysiology of AD and predict the disease progression. A study by DeSimone *et al.* found that positive plasma Aβ42/40 was associated with an increase in extracellular FW in multiple brain regions (DeSimone et al., 2024). Another study found that a lower FW within the limbic network and the default mode network attenuated the impact of tau pathology on memory decline (Qiu et al., 2024). In addition, Schumacher *et al.* found that compared with the healthy control group, the FW in the basal nucleus of Meynert-lateral cortical pathway was increased in the dementia group and the MCI group. This indicates that excessive extracellular free water may be an early indicator of fiber degeneration (Schumacher et al., 2023). Some studies have found that an elevated FWF in the basal nucleus of Meynert and the hippocampus is sensitive to early MCI, and the FW in the hippocampus is more sensitive to the early stages of cognitive decline than the hippocampal volume. This suggests that compared with gray matter atrophy, the FW in the hippocampus may be a more effective early marker of neurodegeneration (Ofori et al., 2019; Chu et al., 2022). In addition, the alteration of FW may be related to neuroinflammation. The neuroinflammation had been proved as a potential essential player in the path-mechanisms of neurodegenerative disease (Balistreri and Monastero, 2023). Recent research had provided additional evidence by demonstrating a substantial correlation between inflammatory markers and free water levels in brain areas (Wu et al., 2024). During the pathology of AD, the increase of cortical free water was associated with the breakdown of myelin cell membranes and cellular components which limited the mobility of water molecules, and was strongly associated with the neuroinflammation of blood–brain barrier (Ji et al., 2019; Nakaya et al., 2022).

Our study observed that in the early clinical stage of AD, the alterations in free water (FW) of the cBF were not associated with cognitive performance. However, in the clinical stage of AD, the FW alterations in cBF were related to overall cognition, mainly involving visuospatial/executive and orientation functions. Interestingly, previous research has found that in PD patients, the FW in the cholinergic basal forebrain mediated the acetylcholine-attention/working memory/executive function relationship, while the cholinergic basal forebrain volume mediated the acetylcholine-temporal region—memory relationship (Crowley et al., 2024). Both this previous finding and our study suggest that different structures and functions of cBF may affect different cognitive functions. Animal studies found that damage to the basal forebrain cholinergic nucleus affected the rat directional force, and this result is consistent with our findings in AD patients (Scattoni et al., 2005). Similarly, another study supports our results, which have revealed that the white matter cholinergic pathway is associated with decreased cognitive function (Park et al., 2015), especially in impaired executive function and visual spatial function (Mesulam, 2013). Previous studies have also found that different sub-regions of cBF are associated with different cognitive functions. The Ch1 region played a crucial role in learning and memory processes by sending cholinergic projections to the hippocampus and regulating neural excitability as well as *θ*-wave oscillation (Gallagher et al., 1995; Hasselmo, 2014). The lesion in the Ch2 region had been shown to interfere with the retrieval function of memory, highlighting its importance in hippocampal-dependent memory (Hall et al., 2014). Although the Ch3 region had been relatively less explored, its connection to primary sensory cortex suggested its role in processing sensory information, particularly with specific projections to primary somatosensory, auditory or visual cortex depending on the distribution of caudal neurons (Chaves-Coira et al., 2018). The Ch4 region, as the main supplier of cholinergic dominance of the cerebral cortex, played a central role in regulating cognitive function through communication with limbic structures and the entire neocortex (Mesulam et al., 1983; Mesulam, 2013). The functional connectivity of the fractional FW within the Ch4 region may reflect the optimization mechanism of the cholinergic system on visuospatial and executive functions. Specifically, acetylcholine enhances spatial representation by reducing the facilitatory interactions between neuronal receptive fields, thereby improving the ability of perceptual discrimination in complex environments (Gratton et al., 2017). It stabilizes the representation of working memory via the prefrontal-striatal pathway, supporting executive control in higher-order cognitive tasks (Harel et al., 2013). Meanwhile, cholinergic signals promote the efficiency of attentional orientation by regulating the fronto-parietal network, enabling the efficient allocation of cognitive resources to behaviorally relevant stimuli (Störmer et al., 2012). The above mechanisms collectively suggest that the FWf in the Ch4 region can serve as the neural basis for integrating spatial precision, executive stability, and attentional flexibility, rather than acting at the early sensory processing stage (Münte et al., 1989). Furthermore, there was important evidence of the cortical cholinergic system in visuospatial attention in parietal cortex. For example, cholinergic receptors in the large parietal cortex were involved in the attentional process (St Peters et al., 2011). Cholinergic input in the prefrontal cortex was associated with memory-related enhanced attention through interaction with the dopamine system (St Peters et al., 2011; Silvestrini et al., 2023). Furthermore, it has been shown that the basal forebrain cholinergic system associated with delayed recall performance, and cholinergic receptor antagonists might influence their effects (Teipel et al., 2015). Our study may additionally suggest that in the early stage of AD, although the cBF is affected without influencing the cognitive level, there may be compensation from other cholinergic pathways. However, further research is needed for verification.

The negative correlation between age and FWf in the Ch1-3/Ch4 regions aligns with evidence that aging accelerates cholinergic system degeneration, marked by volumetric loss in anterior basal forebrain nuclei (Richter et al., 2022) and reduced cortical acetylcholine efficiency (Hampel et al., 2018). Such decline may disrupt synaptic integration within FW networks, impairing their capacity to mediate visuospatial and executive functions. Furthermore, aging alters the neuromodulatory dynamics of acetylcholine, as seen in diminished pharmacological responsiveness (Cammarata and De Rosa, 2022), suggesting that age-related FW reductions reflect both structural atrophy and functional desynchronization.

The ROC curve analysis depicted in Figure 5 highlights the diagnostic potential of the FWf within cBF for identifying patients with AD and aMCI. The AUC values for distinguishing patients with AD from the NC were notably high, particularly in the L_Ch1-3 and R_Ch1-3 regions, with combined AUC of 0.958, suggesting excellent diagnostic accuracy. For aMCI versus NC, the AUC values were moderate, indicating a detectable ability to discriminate early cognitive impairment. Despite lower AUCs in bilateral Ch4 regions for the AD versus the aMCI, the combined AUC still achieved moderate diagnostic value, suggesting that FWf within cBF can be a useful tool for diagnosing AD, this may be related to the fact that aMCI serves as a prodromal stage of AD, with a certain continuity in FWf within the cBF between the two conditions, but with differences in severity, leading to a less pronounced diagnostic effect.

The main advantage of this study lies in being the first to explore the neuroinflammatory responses in different cBF sub-regions in AD and aMCI, and to distinguish cognitive domains to further clarify the impact of cBF on different cognitive domains in AD. However, our study also has certain limitations. First, our study did not include subjects in the pre-clinical stage of AD. Thus, we were unable to further explore whether damage to different cBF sub-regions occurs at an earlier stage. In the future, it would be possible to include populations with subjective cognitive decline or AD gene mutation carriers for further research. Second, our study is a cross-sectional study with a relatively small sample size, which makes it impossible to further explore the dynamic impact of cBF on different cognitive domains as the disease progresses. Follow-up work will increase the sample size and conduct follow-up studies on the subjects. Third, some patients may be taking cholinesterase inhibitors, which may affect the assessment of the cholinergic system. However, previous studies have found that the use of cholinesterase inhibitors has no significant impact on the microstructure and function obtained by DTI (Schumacher et al., 2023).

## Conclusion

In conclusion, our research provides further insights into the neuroinflammatory changes in cBF and its sub-regions in the AD continuum, indicating that an increase in FW in cBF can occur in the early clinical stage of AD, gradually increases with disease progression, and gradually becomes related to cognitive performance. We have also revealed that the neuroinflammatory response in the Ch4 sub-region of cBF is mainly associated with the visuospatial/executive and orientation functions in AD. Our study offers more in-depth insights and knowledge regarding the role of cBF in the mechanisms of AD progression and cognitive changes, potentially providing new evidence for anticholinergic therapies to improve AD cognitive performance.

## Data Availability

The original contributions presented in the study are included in the article/supplementary material, further inquiries can be directed to the corresponding authors.

## Footnotes

^1^ https://fsl.fmrib.ox.ac.uk/fsl

^2^ https://dipy.org/
